# How can pilot work optimally inform surgical RCTs? A review of current evidence

**DOI:** 10.1186/1745-6215-16-S2-P17

**Published:** 2015-11-16

**Authors:** Katherine Fairhurst, Kerry Avery, Elaine O'Connell Francischetto, Chris Metcalfe, Jane Blazeby

**Affiliations:** 1 University of Bristol, Bristol, UK

## Background

Medical Research Council guidance for evaluating complex interventions like surgery, recommends that pilot work should precede randomised controlled trials (RCTs). Methodological considerations pilot work could address include intervention complexity and adherence, education of surgeons on research methodology and recruitment, logistics of co-ordination and feasibility of collaboration. Presented are findings of an ongoing literature review exploring how pilot work may optimally inform surgical RCTs.

## Methods

PubMed was searched for articles with ‘pilot studies’ in the title (inception-03/04/13). Relevant papers and reference lists were reviewed to identify and refine pilot work terminology. Searches combining terms for RCTs and all types of pilot work were conducted in the top 10 medical journals and the journal ‘Trials’ (01/01/11-31/08/13).

## Results

Searches identified 300 abstracts. Randomised pilot studies were described in 84(28.0%), 16(19.0%) of which were non-pharmaceutical pilots, including 5(6.0%) surgical pilots. Of these, only 2 inferred proceeding to a main trial and provided informative recommendations about conduct. Decisions about main trial feasibility focused largely on safety and/or efficacy issues in two other studies.

## Conclusion

The need for improved pilot work in surgical RCTs is recognised, but research into how to conduct it effectively and informatively is scarce, and methodological considerations are rarely addressed. Further planned work, includes analyses of the NIHR database of trial protocols, and exploring perceptions of trialists and surgeons involved in pilot work. This seeks to identify designs and methodologies associated with successful progression to main trials and consequently develop recommendations for the optimal design of pilot work for surgical trials.

